# Affective Bias Through the Lens of Signal Detection Theory

**DOI:** 10.5334/cpsy.58

**Published:** 2021-04-26

**Authors:** Shannon M. Locke, Oliver J. Robinson

**Affiliations:** Laboratoire des Systèmes Perceptifs, Département d’Études Cognitives, École Normale Supérieure, PSL University, CNRS, 75005 Paris, France; Institute of Cognitive Neuroscience, University College London, London, UK; Research Department of Clinical, Educational and Health Psychology, University College London, London, UK

**Keywords:** affective bias, mood disorders, anxiety disorders, signal detection theory, decision-making

## Abstract

Affective bias – a propensity to focus on negative information at the expense of positive information – is a core feature of many mental health problems. However, it can be caused by wide range of possible underlying cognitive mechanisms. Here we illustrate this by focusing on one particular behavioural signature of affective bias – increased tendency of anxious/depressed individuals to predict lower rewards – in the context of the Signal Detection Theory (SDT) modelling framework. Specifically, we show how to apply this framework to measure affective bias and compare it to the behaviour of an optimal observer. We also show how to extend the framework to make predictions about bias when the individual holds incorrect assumptions about the decision context. Building on this theoretical foundation, we propose five experiments to test five hypothetical sources of this affective bias: beliefs about prior probabilities, beliefs about performance, subjective value of reward, learning differences, and need for accuracy differences. We argue that greater precision about the mechanisms driving affective bias may eventually enable us to better understand the mechanisms underlying mood and anxiety disorders.

## Introduction

Affective bias is a clinically-relevant behavioural bias in individuals with mood and anxiety disorders. The bias, usually presenting a diminished response towards reward or away from punishment, can even be measured with simple perceptual-discrimination tasks in the laboratory (e.g., Aylward, Hales, Robinson, & Robinson, 2019; Pizzagalli, Jahn, & O’Shea, 2005). At the same time, sophisticated methods have been developed to understand behaviour in perceptual tasks, namely Signal Detection Theory (SDT) (Green & Swets, 1966), however clinical studies have yet to fully leverage the power of this framework. Here we present a opinion paper which aims to provide the tools and understanding for researchers studying psychopathology to implement SDT to its full potential and suggest a few experiments that we consider informative with respect to affective bias.

We argue that there are two main advantages that SDT has to offer. The first is in the measurement process, as SDT can isolate measures of bias and perceptual sensitivity which jointly affect how the participant responds in the task. Isolating which specific measure of bias correlates with core symptoms (e.g., depression symptom scores) enables a more mechanistic understanding of the causes of these symptoms. The second advantage is being able to model the ideal observer, a theoretical benchmark of optimal performance. SDT is able to incorporate information about the decision scenario, such as reward contingencies, to determine the bias that would lead to the most rewards in the long run. This is particularly useful because any distortions the individual has about the decision scenario (e.g., the perceived value of rewards) can also be converted into predictions about bias by exchanging the true decision context with the perceived decision context in the SDT model. Thus, with SDT, it is simple to compare measured bias, ideal bias, and predicted bias on a per-participant basis. Often when ‘affective bias’ is evoked for mood and anxiety disorders it is done so in a theoretically vague manner. Being more precise about the underlying causes and manifestations of bias enables us to better understand the underlying pathology. Of course, there are many other mood and anxiety symptoms that are better captured by different models, but SDT is uniquely situated to explain biases on simple affective decision making tasks.

In this paper we first provide a broad summary of affective bias and the SDT framework aimed at individuals unfamiliar with this approach. Then we relate this to previous work on affective bias, showing how SDT and optimality considerations can be applied. Finally, we propose future directions that leverage the predictive power of SDT to test whether any of the various documented sources of bias in healthy populations (Rahnev & Denison, 2018) are predictive of affective bias. Together, increasing measurement quality and better understanding the cause for the bias have the potential to improve the understanding of the mechanisms underpinning affective bias.

## Background

### Affective Bias

Affective bias is a broad term that encompasses cognitive or behavioural bias towards (or away from) rewards or punishments. Such bias forms an important part of the aetiology of a wide range of mental health problems (Roiser, Elliott, & Sahakian, 2011). Mood and anxiety disorders, for instance, are thought to involve substantial ‘negative’ affective biases, whereby an individual’s cognitive processes (e.g. perception, attention or learning) are biased towards punishing stimuli and away from rewarding stimuli (Halahakoon et al., 2020; Pizzagalli et al., 2005; Robinson & Chase, 2017). This bias promotes negative mood (e.g. if you are biased to learn and remember negative things that happen to you, it makes your overall mood more negative), which in turn further promotes the underlying negative bias, and can eventually lead to a pathological state (Beck, 1964). These biases therefore form a key part of the diagnosis of mood disorders; where bias away from reward, for example, is encompassed within the diagnostic criterion of “diminished interest or pleasure” (American Psychiatric Association, 2013). Indeed individuals at risk of clinical diagnosis demonstrate elevated negative biases (Rock, Roiser, Riedel, & Blackwell, 2014; Roiser et al., 2011), and longitudinal work suggests that negative biases precede the onset of subsequent diagnoses (Kilford et al., 2015), which suggests a causal role for negative bias in driving clinical vulnerability. Whilst the presence and importance of such biases in mental health has been established for many decades (Beck, 1964), their underlying causes and mechanisms are far from clear (Robinson & Chase, 2017).

### Optimal Decision Making

One way to understand bias is to frame it in the context of optimal decision making. Optimal decision making involves the selection of the best possible choice taking into account the choice context (Berger, 1980). Choice contexts typically have two salient features that need to be considered: the *prior* probability of possible states of the world and the rewards or losses for selecting or not selecting each choice, often referred to as *payoffs*. For example, when a doctor is deciding on a cancer diagnosis for a patient, they may consider other aspects of the patient’s history to gauge the prior probability of developing cancer (e.g., smoker/non-smoker) in addition to the medical results they have just obtained. They should also consider the payoffs of different choice and outcome pairings. For the doctor, these could be: correctly detecting cancer early is good for survival chances but incorrectly diagnosing cancer is stressful and costly for the patient, whereas correctly identifying there is no cancer relieves stress but incorrectly telling the patient they are cancer-free can be deadly.

Once the priors and payoffs are known, the best possible choice can be made by selecting the choice alternative that maximises the expected reward. Many real-world cases have complex rewards and losses that are difficult to quantify, like in our medical example, but in economic or laboratory settings, choice outcomes can be expressed in terms monetary or points-based payoffs. For example, consider a lottery game with two choices, *A* and *B*, where selecting *A* gives a 25% chance of winning a reward of 4 pts versus selecting *B* which gives a 75% probability of winning 2 pts. The expected gain of selecting lottery *A* is 1 pt (0.25 × 4) and for *B* is 1.5 pts (0.75 × 2). Thus, the optimal decision would be to select lottery B because 1.5 pts is greater than 1 pt. In our medical scenario, we cannot usually make such a precise calculation, but weighing up the relative risk of death (extremely high cost) versus causing some mental and financial stress (lower cost), the doctor might err towards a positive diagnosis in an ambiguous case. Optimality can be considered in numerous modelling frameworks, including SDT which we make use of here.

### The Signal Detection Theory Framework

Signal Detection Theory (SDT) is a well established framework for modelling perceptual decision making (Green & Swets, 1966). Perceptual decision making is based on noisy information reaching our senses that is then stochastically represented in the brain. Both of these factors add an additional layer of uncertainty in the decision process. Returning to our medical example of a doctor deciding on a cancer diagnosis, the information the doctor receives can be corrupted by noise in various ways, such as the visual blur in an x-ray image or neuronal noise involved in the sense of touch when inspecting a lump. The SDT framework is flexible in that can be applied to most scenarios of repeated perceptual discrimination or categorisation, such as reporting whether stimuli have rightward motion or leftward motion or categorisation in the laboratory, such as, or a series of cancer/not-cancer diagnoses in the hospital. Furthermore, SDT can be both descriptive, when applied in the context of empirical observations (e.g., quantifying how two doctors differ in how readily they diagnose cancer), or prescriptive, in the sense that it can describe what the optimal decision is for a given scenario.

A feature of the SDT approach, and perhaps its greatest strength, is its ability to dissociate the separate effects of perceptual sensitivity and perceptual bias on the decision making process. That is, the degree of noise in the sensory evidence vs. the observer’s propensity to choose one choice alternative over the other. Once the bias measure, the decision criterion, has been isolated, it can then be easily compared to the optimal decision criterion as well as the bias of other observers, taking into account any differences in sensitivity, as we will demonstrate next. Thus, the SDT framework is a powerful tool for measuring and contextualising behavioural biases.

### Applying The SDT Model

**Figure 1:**
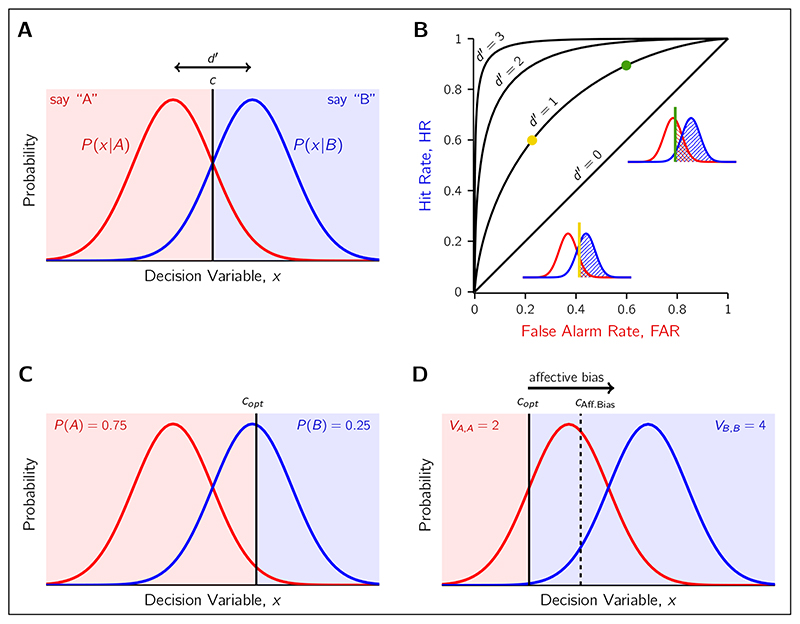
Illustration of the Signal Detection Theory (SDT) framework. **A)** The observer makes a measurement, *x*, of the stimulus, either stimulus *A* or stimulus *B*. The probability of a measurement, conditioned on the stimulus, is assumed to be Gaussian distributed due to the effect of sensory noise. The observer selects a decision criterion, *c*, that provides a mapping from *x* to a response (e.g., say “A”). Perceptual sensitivity, *d*′, is reflected in the separation of the curves. **B)** Each pair of *d*′ and *c* predict a unique combination of Hit and False Alarm rates. Curves reflect all the possible Hit rate and False Alarm rate combinations for a given perceptual sensitivity (e.g., *d*′ = 1). Two example criterion placements (green and gold) are shown on the curves and the SDT model insets. The green criterion setting leads to more Hits and False alarms (shaded regions) compared to the gold criterion setting. **C)** When priors are not equal for the two choice alternatives, the optimal criterion, *c_opt_*, is no longer at the neutral location centred between the two measurement distributions. As shown, stimulus *A* is more probable than stimulus *B*, causing a rightward shift in *c_opt_*. Consequently, stronger evidence of *B* is needed to report “B”. **D)** Similarly for unequal payoffs. Correctly guessing *A* receives 2 units of reward, whereas correctly guessing *B* receives 4 units. This shifts *c* *_opt_* leftward so that stronger evidence of *A* is needed to report poorly rewarded “A”. In an unequal reward context, affective bias is an increased preference to report the low-reward option (here “A”). This can be modelled as a shift in *c* towards the high-reward distribution. An example of a criterion influenced by affective bias is shown by the dashed line.

The standard SDT model describes the decision-making process for when one of two potential sensory stimuli, *A* and *B*, is presented to an observer and they are asked to report which they perceived. The presentation of stimulus *A* does not produce the same sensory response in the observer each time it is shown, due to the various sources of noise corrupting the signal. The same is true for *B*. Instead, we refer to the noisy measurement of the stimulus in the observer’s brain as *x*, the value of which is unobserveable to the experimenter. An assumption of the standard SDT model is that the sensory noise affecting *A* and *B* is Gaussian, with the conditional measurement distributions *p*(*x*|*A*) and *p*(*x*|*B*) modelled as two equal-variance Gaussian distributions (see Figure 1). These distributions represent the values of *x* one can expect to observe for repeated presentations of identical *A* and identical *B*.

The *perceptual sensitivity* of the observer is reflected in the separation of these distributions in units of SD,

(1) $$d^{\prime }=\frac{\mu_{B}-\mu_{A}}{\sigma }$$

and is referred to as *d*′ (“dee-prime”). A common modelling choice, which we adopted for this paper, is to rescale the decision axis by a factor of *1/σ* so the variance of *p*(*x*|*A*) and *p(x|B)* is 1. This allows *d*′ to be read directly as the distance between the distributions because the re-scaling takes care of the denominator in Eq. 1. Consequently, when sensory noise is higher, the measurement distributions are closer together, and perceptual sensitivity (*d*′) is lower. This is intuitive: from the observer’s perspective, they must infer if their measurement *x* came from the *p*(*x*|*A*) or the *p*(*x*|*B*) distribution, which are more easy to confuse if the distributions are closer together. If the decision axis is unscaled the confusion resulting from increasing noise can be thought of in terms of simply a greater overlap in the two conditional distributions.

To convert the noisy measurement *x* into a categorical response “A” or “B”, the observer must place a decision *criterion*, *c*. This criterion divides the decision space into two regions: values of *x* that lead to “A” responses and those that lead to “B” responses. The criterion can be placed at any point along the decision axis, and is independent with respect to the perceptual sensitivity. The insets in Figure 1B shows two examples of criterion placement for an observer with a *d*′ = 1, and the consequent change in response probabilities. By shifting the criterion to the left, the observer is more likely to respond “B” correctly for *B* stimuli, but also incorrectly “B” for *A* stimuli. In fact, it is these two response probabilities, referred to as “Hits” and “False Alarms” respectively, that are used to calculate the observer’s empirical *d*′ and *c* from a series of judgements. We calculate

(2) $$d^{\prime }=z(\text{HR})-z(\text{FAR})$$

and

(3) $$c=-\frac{1}{2}[z(\text{HR})+z(\text{FAR})]$$

where HR is the hit rate, FAR is the false alarm rate, and *z* is the normal inverse function. The curves in Figure 1B, referred to as *receiver operating characteristic* curves, show the resulting unique pairs of HR and FAR for different combinations of *d*′ and *c*.

Finally, the decision criterion can also be converted to the more general *β* representation, which is independent of perceptual sensitivity, through the following relationship:

(4) $$\ln\beta =cd^{\prime }$$

for a fairer comparison across observers or tasks where *d*′ may differ. Either ln *β* can be compared, or the equivalent criterion for a *d*′ can be compared by solving for *c*.

### Optimality In SDT

For a given perceptual sensitivity, there is only one location of the decision criterion that is optimal to maximise rewards. This theoretical benchmark is often referred to as the *ideal observer*, who makes the best decision possible given the noisy sensory input (Green & Swets, 1966; Tanner & Birdsall, 1958). The optimal criterion, *c_opt_*, depends on both the prior probability of *A* and *B* as well as payoff structure of the environment (see Figure 1C-D):

(5) $$C_{opt}=\frac{\ln\beta_{opt}}{d^{\prime }}$$

where

(6) $$\beta_{opt}=\frac{P(A)}{P(B)}\frac{V_{A,A}-V_{A,B}}{V_{B,B}-V_{B,A}}.$$

*P(A)* and *P(B)* are the prior probabilities of each stimulus. The outcomes for four possible stimulusresponse pairs have the associated payoff *V_r,s_* for responding *r* to stimulus *s* (rewards are positive; losses negative). If one alternative is more probable or is rewarded more highly, the ideal observer will shift their criterion from the neutral position (*c_neu_* = 0) so more responses will be of that category (see Figure 1C-D). This is equivalent to requiring less sensory information consistent with that category to select it. When both priors and payoffs are asymmetric, then their effects on the optimal criterion are additive (Stevenson, Busemeyer, & Naylor, 1990).

Biases in perceptual decision making can be quantified by comparing where the observer chose to place their criterion relative to the optimal criterion, in a manner that takes into account the perceptual sensitivity of the observer. For example, one way a ‘negative’ affective bias might emerge is by moving the criterion towards the highly rewarded option relative to the optimal (e.g. a criterion rightward of *c_opt_* in Figure 1D), leading the individual to choose the low-reward option more frequently than they should.

Any deviation from the optimal criterion is classified as *suboptimal* decision making, and *conservatism* is the term used to specifically refer to biases where *c* is placed between *c_opt_* and *c_neu_* at 0 (Green & Swets, 1966). For example, the affective bias criterion in Figure 1D is an instance of conservatism. In fact, it is common to find that normal healthy observers are conservative in criterion placement (Ackermann & Landy, 2015; Ulehla, 1966), and a wide range of explanations for the conservatism have been proposed (Rahnev & Denison, 2018). It is for this reason we suggest investigating whether these potential sources of suboptimality are predictive of affective bias, given that they share the same behavioural signature. We detail these sources of suboptimality and our proposed tests to link them to affective bias in the Future Directions section.

### Interim Summary

The SDT framework allows researchers to isolate and quantify the perceptual sensitivity and perceptual bias of an observer and directly compare choice behaviour to optimal decision-making. As affective bias can manifest as a suboptimal propensity to choose the low reward outcome, as shown in the following examples from previous clinical studies, we propose that the SDT framework is ideal for quantifying affective bias in perceptual tasks and comparing amongst observers with potentially different perceptual capabilities. Furthermore, existing hypotheses about conservative criterion placement could be leveraged to investigate the source of affective bias in decision making in future research.

## Using SDT To Understand Affective Bias

In this section we review two approaches that have been used to measure affective bias with perceptual tasks. The first is a tone-discrimination task of Aylward, Hales, et al. (2019), which we re-analyse in the SDT framework. The second is a variant on the standard signal-detection task that uses a probabilistic reinforcement schedule. Following the SDT principles we outlined above, we show how to investigate optimality in both scenarios.

### Tone Discrimination Task

**Figure 2:**
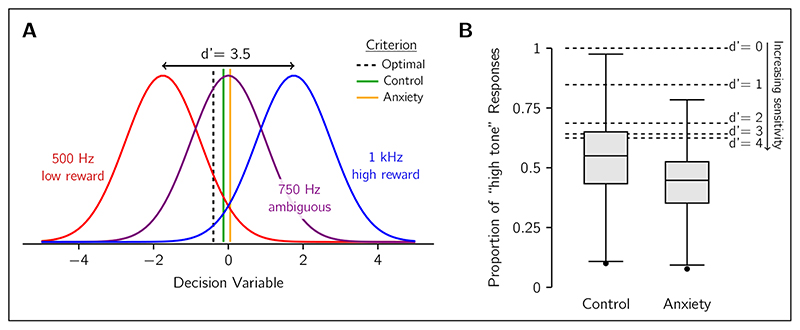
The tonediscrimination task of Aylward, Hales, et al. (2019). A) Participants reported if they heard the low-frequency tone (red) or the high-frequency tone (blue). The reward for correctly guessing the high-frequency tone was four times larger than for the low-frequency tone. On some trials, an ambiguous tone (purple) was played and was rewarded randomly. The graph illustrates the SDT model with the expected probability distributions of the decision variable for each of the tones. Example shown for an observer with a perceptual sensitivity of *d*′ = 3.5 (i.e., the sensitivity of the average anxiety-group participant). The optimal decision boundary (dashed) provides the decision rule that maximises expected reward: choose “low tone” if the decision variable is lower than this value, and “high tone” if it is above. Critically, this boundary does not align with the midpoint between the red and blue distributions (peak of the purple distribution) because it is optimal to have a bias for responding with the more highly rewarded “high tone”. Average criterion for control group (green) and anxiety group (gold) are also shown, demonstrating a conservative shift away from optimal, and an even greater shift for the clinical group interpreted as affective bias. **B)** Boxplots of the empirical distributions of the proportion of “high-tone” judgements for the ambiguous stimulus, split by test population. The lower proportion of high-tone responses of the anxiety group is the affective bias effect (*p* = 0.003, BF_10_ = 12.51; Aylward, Hales, et al., 2019). Horizontal dashed lines indicate the expected proportion of responses for the ideal observer who uses the optimal decision boundary. The expected proportions are shown for several perceptual sensitivities ranging from no sensitivity (*d*′ = 0) to near-perfect sensitivity (*d*′ = 4).

To illustrate affective bias in the context of optimal decision-making using SDT, we consider the response of human participants in the tone-discrimination task of Aylward, Hales, et al. (2019). This study recruited participants that were either experiencing clinically significant low-mood and/or anxiety symptoms or were asymptomatic controls. They discriminated between a high tone (1 kHz), which was highly rewarded when correctly identified, and a low tone (0.5 kHz), that yielded four times less reward (note that stimulus/outcome randomisation was added in later versions of the task; Daniel-Watanabe, McLaughlin, Gormley, & Robinson, 2020). Occasionally, an ambiguous intermediate (0.75 kHz) tone was played and was randomly treated as a low or high tone for scoring purposes including the magnitude of reward (i.e., half the trials a low-tone response was required and furnished the small reward value, and the other trials a high-tone response was required and was rewarded well). Figure 2A shows the SDT diagram for this experiment, with three distributions for each of the three possible tones. Participants had overall high discrimination performance between low and high tones, reflecting high perceptual sensitivity. The mean *d*′ for the control and anxiety groups were 3.6 and 3.5 respectively. Using Eq. 4 to compute the equivalent criteria for *d*′ = 3.5, the average criterion was –0.13 for the control group and 0.05 for the anxiety group (see Figure 2A), both of which are closer to neutral location at 0 than the optimal at –0.4.

To maximise reward, the participant should be biased towards responding “high tone” because it is more highly rewarded. This can be seen in the criterion (dashed line) depicted in Figure 2A, which is shifted leftward from the neutral position at 0. The bias should be most the evident for the ambiguous trials, where the proportion of “high-tone” to “low-tone” responses is a good indicator of criterion placement. The dashed lines in Figure 2B show the expected proportion of “high-tone” judgements in the ambiguous condition for ideal observers with several different levels of perceptual sensitivity. The optimal criterion is affected by sensitivity (see Eq. 1): the higher the sensitivity, the more the observer should trust the sensory measurement and consequently lowering the bias from reward. Yet, even for extremely high perceptual sensitivity, it is still optimal to have a bias for the more rewarded stimulus.

Participants in this task, however, showed a wide range of biases when presented with the ambiguous stimulus. On average, the control group responded “high tone” more often than the anxiety group. But many of them, as well as, critically, more than half of the anxiety group, showed a preference for selecting the low-rewarded “low-tone” option, interpreted as an affective bias. This can be seen in the average placement of the criterion by each group (green and gold lines in Figure 2), with the anxiety group placing their criterion further from the optimal location than the control group. We also note that using a log-space representation, typical for auditory experiments of tone-frequency discrimination (to match the encoding characteristics of the cochlea), does not change the interpretation of the results and in fact predicts overall higher proportions of “high-tone” responses.

Despite being able to apply SDT to re-evaluate this previous study, we note that the design of this task (optimised for translational use) is not ideal for using the SDT method. First, the use of three stimuli, with two being obvious and the other ambiguous, is not necessary. To measure *d*′ and criterion, only two stimuli are necessary, as illustrated by the graphs in Figure 1. Adding a third stimulus makes the fitting the SDT model more complex, particularly if it has a random reward contingency. Additionally, when participants have very high levels of performance as with this example task, the quality of the *d*′ and criterion measurements is reduced. Trials in which the participant selects the wrong option are very important for fitting the SDT model, and wrong answers occur less frequently when the task is easy. The optimal criterion is also much closer to neutral when *d*′ is large, which could lead to smaller differences in the criterion between the clinical and control groups (e.g., the criterion difference in Figure 2A). As such, we recommend using tasks with two stimuli that are somewhat difficult to tell apart (e.g., *d*′ *≈* 1) to optimise the task for the SDT approach. In summary, while this example task shows how SDT may be applied, it also highlights that experiment design is an important factor to ensure good measures of sensitivity and bias, as well as maximising the opportunity to observe differences between clinical and healthy populations.

### Probabilistic Reward Tasks

A series of clinical studies (e.g., Der-Avakian, D’Souza, Pizzagalli, & Markou, 2013; Pizzagalli, Iosifescu, Hallett, Ratner, & Fava, 2008; Pizzagalli et al., 2005), have employed a set of perceptual tasks called Probabilistic Reward Tasks (PRTs) (McCarthy & Davidson, 1979) with SDT measures. PRTs are specifically adapted for reinforcement learning by varying only the reinforcement schedule probabilistically to induce a preference for reporting one stimulus over the other (i.e., priors and payoffs are the same for the two stimuli). For example, rewards are only given for a subset of correct trials, and they are distributed unevenly for correct *A* responses versus correct *B* responses. In contrast, tasks using the standard signal-detection theory approach like we have described, provide reward deterministically for every response and vary the value of reward to induce the response bias. A major finding using PRTs is that the degree of bias towards the more frequently reward stimulus is reduced with elevated depressive symptoms (i.e., affective bias Pizzagalli et al., 2008, 2005).

Unlike in the Aylward, Hales, et al. (2019) study above, the researchers using the PRT separate out the individual components of sensitivity and bias metrics functionally similar to the *d*′ and *c* values defined here. However, the approach taken with these PRTs fall short of the approach proposed here in that it does not consider optimality. To see this, we can rephrase Eq. 6 for a probabilistic reinforcement schedule as follows (ignoring the specific response and feedback history):

(7) $$\beta_{opt}=\frac{P(A)}{P(B)}\frac{R_{A}V_{A,A}-V_{A,B}}{R_{B}V_{B,B}-V_{B,A}}=\frac{R_{A}}{R_{B}}$$

The terms *R_A_* is the probability of being rewarded for correctly identifying stimulus *A*, determined by the reinforcement schedule (e.g., 30/50 A’s rewarded: *R_A_* = 0.6), and similarly *R_B_* for stimulus *B*. The equation simplifies the prior odds ratio because it is typically 1, the value of making an incorrect choice because it is typically 0, and the value of correct choice is dropped because equal reward values are typically used (*V*_*A*,*A*_ = *V*_*B*,*B*_).

Following this logic, the participant’s bias in a PRT should stabilise after a sufficient learning period and the degree of bias considered optimal should vary according to the perceptual sensitivity of the participant and the reinforcement schedule. Yet, to our knowledge, optimality has not been considered in PRT studies of affective bias. However, any of the recommended experiments proposed below could be implemented with the standard signal-detection task or a PRT. For investigating optimality, we would recommend using the standard version and giving participants sufficient training in the task, to reduce complexity and reduce effects of the reinforcement history on behaviour. For investigating learning, we would recommend the PRT task (and for further reading on our thoughts about the role of reinforcement learning in mood and anxiety disorders please see Aylward, Valton, et al., 2019; Mkrtchian, Aylward, Dayan, Roiser, & Robinson, 2017; Robinson & Chase, 2017).

## Directions for Future Research

Affective biases have been long known to play a critical role in promoting and maintaining core symptoms of mental ill health. While biases in general are not unusual in perceptual tasks (Ackermann & Landy, 2015; Rahnev & Denison, 2018; Ulehla, 1966), investigating the source of the bias responsible for divergent behaviour between clinical and healthy populations could lead to a more precise understanding of the underlying cognitive processes maintaining affective bias. Criterion biases can be a behavioural signature of one or more processes influencing the decision making process, generally related to how the individual subjectively experiences the choice context or their own behaviour. This is because correctly calculating the optimal criterion requires three pieces of knowledge: the prior probabilities of each stimulus, the payoff structure of the environment, and perceptual sensitivity of the observer. If the observer is incorrect in their beliefs for one or more of these components, then they may set their criterion suboptimally. As such, narrowing down which of these causes drives the increased bias in mood and anxiety disorders can provide us with more precise targets for treatment intervention. In this next section we suggest a number of future directions to clarify the underlying cause of biased decision making behind affective bias from a SDT perspective. We take the approach of using secondary measures to probe how the individual perceives the choice context or their own performance to build a predictive participant-specific SDT models. We aim to find results in these secondary measures that predict greater bias and match well with the choice behaviour of the participants.

### 1 Beliefs About Prior Probabilities

**Figure 3:**
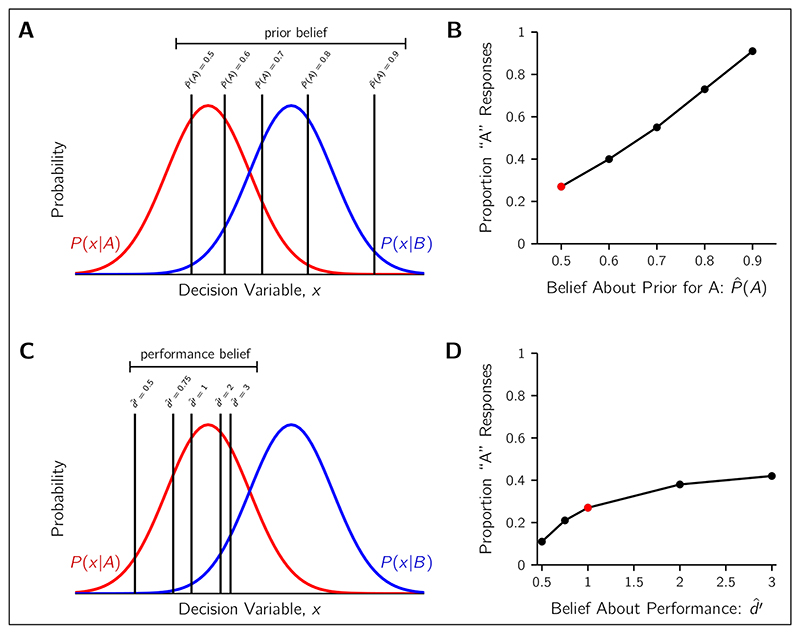
Predictions for criterion placement and proportion of low-reward responses for different incorrect beliefs. Predicted results shown for low-reward stimulus A (red) and high-reward stimulus B (blue), with equal priors (*P(A) = P(B)* = 0.5), correct B responses being rewarded twice as much as correct A responses (*V_A,A_* = 2 and *V_B,B_* = 4), and perceptual sensitivity of *d*′ = 1. **A)** Criterion placement for different beliefs about the prior probability of stimulus A. The greater the estimated probability of A $(\hat{P}(A)),$ the greater the rightward shift in the criterion. **B)** The proportion of low-reward responses, for different beliefs about the prior probability of A. Prediction for the use of the optimal criterion with correct beliefs shown by the red marker, and incorrect prior beliefs by the black markers. The more the observer believes A is probable, the more low-reward responses. **C)** Criterion placement for different beliefs about perceptual performance. Under-estimations of performance (${\hat{d}}^{\prime }$ < 1) lead to leftward criterion shifts, and over-estimations of performance (${\hat{d}}^{\prime }$ > 1) lead to rightward criterion shifts. **D)** The proportion of low-reward responses, for different beliefs about performance. Predicted proportion with correct beliefs shown by the red marker, and incorrect beliefs by the black markers. Over-estimations of performance lead to more A responses and under-estimations to less.

One compelling hypothesis for affective bias in perceptual tasks is the incorrect beliefs about prior probabilities. One of the key clinical features of depression and anxiety is a belief that bad things are more likely to happen than good things. This is the basis, for example, of negative cognitive schemata that are targeted by cognitive behavioural therapy (Beck, 1964). If the individual believes that low-reward stimuli are presented more often than the high reward stimuli (due to their negative schemata or a focus towards remembering low-reward events and forgetting high-reward events), then it is rational to shift the decision criterion so as to select the low-reward stimulus more often. As illustrated in Figure 3A-B, this bias can offset the optimal shift due to the payoff structure (i.e., move the criterion back towards the neutral location), or, in the case of extreme over-estimation of the low-reward stimulus probability, reverse the bias entirely.

This source of suboptimality has been demonstrated in several tasks with normal, healthy populations. For example, humans can learn the prior probabilities of the choice environment through experience, however this takes time and can be conservatively distorted as if participants thought the priors were closer to 50%–50% (Norton, Acerbi, Ma, & Landy, 2019). Alternatively, information about priors and payoffs can be explicitly conveyed to the observer, but they still may choose to rely on the recent stimulus history for judging the prior probabilities (Yu & Cohen, 2009). Additionally, there is also well-known subjective distortions of probability (Fox & Poldrack, 2009; Kahneman & Tversky, 1979), particularly for the very high or low probabilities, that also affect criterion placement (Ackermann & Landy, 2015). In the clinical domain, previous work has indicated that those with higher trait anxiety make stronger use of prior probabilities (Kraus, Niedeggen, & Hesselmann, 2021), so an incorrect belief is likely to have a large behavioural effect such as the reversal of preference from high-to low-reward stimuli. Thus, incorrect beliefs about priors, either from biased learning from experience or general subjective distortions of probability, can account for both the affective bias signature of showing a degree of conservatism in their responses, as well as showing stronger attraction to the low-reward stimulus than the high-reward stimulus (e.g., as in Aylward, Hales, et al., 2019).

#### ProPosed Prior-beliefs test

To test this hypothesis, patients and controls could be given a task where the prior probability of the stimuli are directly manipulated. For example, in one block of trials the split between the two stimuli could be 30%–70% and the next 60%–40%, and so on to cover a range of possible prior probability asymmetries for low- and high-reward stimuli. Participants would be told at the beginning of each block the probabilities of the stimuli have changed but would be left to work out the ratio themselves. Then, at the end of a block, a secondary measure would probe the perceived prior probabilities; participants would be asked to report the the frequency of low- versus high-reward stimuli (e.g., 45%–55%). These reported priors could be used in several analyses. The first, to assess if the clinical group overestimates the probability of the low-reward more so than the non-clinical group. The second, would be to see how well these reported prior beliefs predict criterion placement. For example, if the prior probabilities for *A* (low-reward) and *B* (high-reward) in a block were 50%–50%, but the observer thought they were 80%–20%, then they incorrectly believe the optimal criterion location is much further to the right (see *P*(*A*) = 0.5 versus *P*(*A*) = 0.8 in Figure 3A). Then, using the pattern of Hits and False Alarms, one can compute the observer’s empirical criterion for that block using Eq. 3. If incorrect prior beliefs are driving affective bias, then the predicted criterion for *P*(*A*) = 0.8 and their empirical criterion should match. Equivalently, one could compare the predicted proportion of *A* responses according to the SDT model (Figure 3B) versus the actual proportion of *A* responses for the incorrect belief of *P*(*A*) = 0.8. That is, after accounting for the incorrect prior beliefs, the individual is otherwise rational in the placement of the decision criterion. If the incorrect priors hypothesis is confirmed, research could then target how the incorrect beliefs are formed from past experiences within the task (Norton et al., 2019; Pizzagalli et al., 2008; Yu & Cohen, 2009).

### 2 Beliefs About Performance

Another incorrect belief that could lead to an affective bias is the observer’s belief about their own performance. Specifically, if an observer believes their performance to be much better than it actually is (i.e., higher perceived *d*′), then we would expect to see conservatism in their criterion placement (Kubovy, 1977). This is because the influence of contextual factors, such as payoffs and prior probabilities, is diminished when the perceptual sensitivity is high, so overestimating their perceptual ability will lead the observer to trust their senses more than they should. Conversely, observers who underestimate their ability should show the opposite pattern, responding more strongly in accordance with the relative value of rewards. These predictions are illustrated in Figure 3C-D, where overestimating *d*′ leads to more low-reward *A* responses than optimal, and underestimating to less *A* responses by shifting the criterion right or left respectively.

Consistent with low self-confidence, which forms a key part of the clinical presentation of deperession (Beck, 1964), previous research shows that individuals with anxiety/depression symptoms tend to report low confidence when performing perceptual tasks (Rouault, Seow, Gillan, & Fleming, 2018). These trial-to-trial reports, if they inform a global sense of belief in performance (Rouault, Dayan, & Fleming, 2019), would predict a bias in criterion placement that is a reverse of affective bias. That is, the observer would be strongly influenced by the reward structure of the task, reporting the high-reward option against their own sensory evidence. This is supported by the findings of strong prior use in individuals with higher trait anxiety (Kraus et al., 2021). It is for this reason that we believe investigating beliefs about overall task performance is important to understanding the interplay between affective bias and metacognitive differences in observers with anxiety or mood disorders. However, how observers come to form an estimate of their own perceptual sensitivity is not well understood and rarely discussed (Rouault et al., 2019; Rouault & Fleming, 2020).

#### ProPosed Performance-beliefs test

Beliefs about performance could be investigated in a simple signal-detection task with a low reward and a high reward stimulus that are presented 50%–50% and without varying difficulty level. Two types of secondary measures could be collected in the same task: local confidence reports, where after each perceptual decision, the observer would report their sense of confidence in the correctness of their decision, and global confidence reports, where at the end of the experiment, the observer would report their belief about their overall performance (e.g., on a scale of 0 to 100% correct). We predict that the anxiety/mood individuals would report lower confidence in their performance both after trials and at the end of the experiment, but they would also report the low-reward stimulus more frequently than optimal (in accordance with affective bias but in conflict with the predictions of SDT). To be precise, the clinical group would have more low-confidence ratings after trials and rate their global performance as lower as compared to the control group, but have an empirical criterion consistent with a larger overestimation of *d*′ than the controls. Understanding how incorrect performance beliefs is important because it could interact with the other sources of bias (e.g., prior beliefs), reducing the degree of affective bias, which need to be modelled to accurately predict choice behaviour.

### 3 Subjective Value of Reward

**Figure 4:**
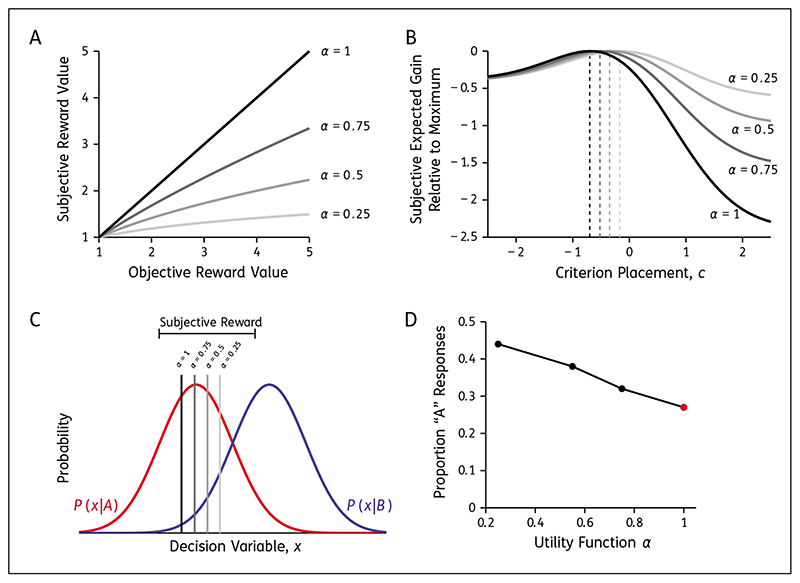
The effect of subjective value on decision making. Predicted results shown for low-reward stimulus A (red) and high-reward stimulus B (blue), with equal priors (*P(A) = P(B)* = 0.5), correct B responses being rewarded twice as much as correct A responses (*V_A,A_* = 2 and *V_B,B_* = 4), and perceptual sensitivity of *d*′ = 1. **A)** Example subjective utility functions. When *α <* 1, the subjective value of the two reward outcomes (A/B) is more similar. **B)** Reward landscape is affected by the subjective utility function. Dashed lines show the criterion placement expected to maximise expected gain with the subjective reward values. When *α <* 1, there is a dampening affect, with the perceived consequence of criterion misplacement being small (i.e., loss incurred is minimal for other values of *c*). **C)** Optimal criterion placement according to the subjective reward ratios. As *α* → 0, the criterion placement that maximises expected reward with these distorted reward values shifts towards the neutral position between the distributions. **D)** The proportion of low-reward responses when criterion is adjusted according to the subjective value of reward. When the subjective value matches objective value (red), the proportion of A responses is lower than if the subjective value is used with *α <* 1 (black).

Another plausible explanation is that the individuals with affective bias value rewards differently. In particular, if there is a devaluing of the high reward, then the subjective value of the low and high reward are more similar, and thus there is less motivation to prefer one outcome over the other. This would be consistent with anhedonia in depression, framed as “loss of interest or pleasure” in diagnostic criteria (American Psychiatric Association, 2013), in which previously enjoyable things lose their value to depressed individuals. This is indeed observed on a wide range of reward-based cognitive tasks (Halahakoon et al., 2020; Pizzagalli et al., 2005). To understand how an individual subjectively experiences reward, scientists can measure the subjective utility function (Fox & Poldrack, 2009; Kahneman & Tversky, 1979), which maps the objective value of a reward, *V*, to its subjective value, *u*(*V*), and is typically expressed as in terms of a power function

(8) $$u(V)=V^{\alpha }$$

(see Figure 4A) with 0 < *α* < 1 typical (e.g., see Farashahi, Azab, Hayden, & Soltani, 2018; Hertwig & Erev, 2009). In the SDT context, changing the subjective utility function has implications for the reward landscape (Figure 4B) and consequently the placement of the decision criterion (Figure 4C). The closer the subjective reward values are to equal, the closer the rational observer will place their criterion to the neutral location (Ackermann & Landy, 2015), because biasing the responses in favour of the high-reward stimulus makes little sense if the difference in reward is small, resulting in fewer high-reward responses (Figure 4D). Given this context, we would hypothesise individuals expressing affective bias have smaller *α* values than a healthy control population if subjective utility is the source of affective bias in perceptual tasks. Clinical support for this hypothesis on subjective value in mood and anxiety disorders is limited and of mixed results (Baek et al., 2017; Charpentier, Aylward, Roiser, & Robinson, 2017; Chung et al., 2017), suggesting further research is required to understand the role of reward value distortions in choice behaviour. Additionally, to our knowledge, subjective reward values and bias in a perceptual task have not been compared within individuals with anxiety or mood disorders.

#### ProPosed subjective-value test

To test this hypothesis, participants would do a simple signal-detection task (e.g., a low reward and a high reward stimulus, presented 50%–50%, constant difficulty level). The secondary measure would be a lottery task to measure the subjective utility functions of participants. This would involve, presenting a series of lottery choices like we described earlier (e.g., 25% chance of winning 10 dollars, or 100% chance of 2 dollars). Then one could assess group-level differences in the subjective evaluation of reward (i.e., differences in *α*), as well as the degree to which subjective value predicts criterion placement in the perceptual task. For example, if a participant has a strong insensitivity to reward according to the lottery task (e.g., *α* = 0.25), then SDT would predict a rightward shift in their criterion in the perceptual task (see Figure 4C) and an increase in the proportion of *A* responses (see Figure 4D). Thus this experiment has the potential to show a direct relationship between subjective processing of reward and behavioural bias.

### 4 Learning the Context

In several aspects, observers must succeed in learning about the context and their own performance to respond optimally. There is extensive clinical evidence for learning differences in depression and anxiety, from general cognitive impairment (Rock et al., 2014) through to more specific changes to learning captured by, for example, reinforcement learning models (Aylward, Valton, et al., 2019; Halahakoon et al., 2020). In perceptual tasks, participants must learn to correctly estimate the prior probabilities of the stimuli and the reward contingencies, as well as monitoring feedback and confidence to estimate perceptual sensitivity. Some models consider this learning process as a gradual adjustment of the criterion to its optimal position (Busemeyer & Myung, 1992; Maddox & Bohil, 2003), where adjustment stops when the rate of reward appears to be no longer increasing (i.e., the maximum is found; peaks in Figure 4B). Others investigate the learning process at the component level (e.g., priors) in dynamic environments with changing choice context (Norton et al., 2019). If learning rates differ between those with and without anxiety/mood symptoms (Aylward, Valton, et al., 2019; Vrieze et al., 2013), this could increase criterion conservatism and cause sluggish responses to change in dynamic environments. Supporting this hypothesis is work by Pizzagalli et al. (2008) showing that clinically depressed individuals have difficulties integrating the reinforcement history over time to correctly bias their responses towards highly rewarded options.

#### ProPosed learning test

To investigate the role of learning in affective bias, one could use the overt-criterion task of Norton et al. (2019) with changing prior probabilities for low- versus high-reward stimulus. In this perceptual task, observers are shown stimuli with high external noise (i.e., experimenter-controlled noise) and are required to continually monitor the stimulus priors to adjust their criterion accordingly. Instead of reporting stimulus *A* or *B*, observers are asked to explicitly place a criterion and their response would be scored as correct if this criterion correctly categorises the stimulus. This modification allows for rapid shift in criterion to be detected and modelled. We predict that observers who display affective bias could either 1) have a slower learning rate, 2) a stronger bias towards equal probabilities, or 3) a bias inflating the probability of the low-reward stimulus. This task would be well complemented by including additional learning tasks, such as the multiarmed bandit task (Aylward, Valton, et al., 2019), to directly compare learning parameters across models of the tasks, as well as the subjective utility measures mentioned in the previous future direction.

### 5 Need for Accuracy

There are two ways in which a need for accuracy could affect criterion placement. The first is by selecting a decision strategy that emphasises getting the perceptual judgements correct over earning the most reward in the task. In the extreme, ignoring the reward values entirely would lead to the highest perceptual decision accuracy, but in practice observers are more likely to select a mixture strategy that is a trade-off between accuracy and gains (Maddox & Bohil, 1998, 2003). This is because there is the tension between maximising gain versus maximising accuracy when payoffs are unequal. It is as if there is a cognitive cost to being incorrect and/or cognitive reward for being correct that is at play in perceptual decision-making.

This trade-off behaviour in strategy selection can also be interpreted as a different need for accuracy. Namely, the need for our sense of confidence to best reflect the true probability of being correct, as sacrificing some gains for better accuracy may foster more accurate judgements of perceptual confidence (Locke, Gaffin-Cahn, Hosseinizaveh, Mamassian, & Landy, 2020). The payoffs of a task can influence confidence ratings (Lebreton et al., 2018; Locke et al., 2020) despite not changing that probability that the decision was correct. For example, this can be an overconfidence when reporting the highly-rewarded stimulus and underconfidence when reporting the low-reward option. As such, the less influence observers give to the payoffs of a task, the more accurate their confidence judgements will be. This hypothesis is supported in the affective bias literature by a phenomenon known as a ‘catastrophic response to perceived failure’ (Elliott et al., 1996; Roiser et al., 2011), where one instance of failure can have a snowball effect for a future series of failures. Thus the consequence of making a judgement against what the observer believes to be correct has a strong psychological cost that has not been factored into the optimal criterion calculation (Eq. 6) by the experimenter. In terms of confidence ratings, individuals with anxious-depression symptoms are also more accurate in their confidence judgements than those with other psychopathologies (Rouault et al., 2018), as would be predicted by this hypothesis.

#### ProPosed need-for-accuracy test

To test this hypothesis, individuals with and without anxiety and/or mood disorder symptoms could perform a perceptual task with the reward values changing from block to block. Strategy could be assessed by asking participants to self-report the importance they placed on getting the answers correct versus earning the most reward at the end of each block. Additionally, confidence judgements, on the correctness of their decision, could be included after every trial. Standard extensions of the SDT framework for confidence would then reveal if that observer let the rewards influence their confidence (Locke et al., 2020). If affective bias is driven by a need for high accuracy, we would see the anxiety/mood disorder individuals giving greater preference for being correct over earning more reward and being more susceptible to letting reward influence their sense of confidence.

### Advantages and Limitations of the Proposed Approach

SDT is a flexible modelling framework that has a lot of advantages. There is the ability to dissociate sensitivity and bias, tailor the model to individual observers, specify the behaviour of an ideal observer, and make quantitative predictions about behaviour for various suboptimalities. This allows researchers to go beyond simply cataloguing suboptimalities, to actually investigating their source (Rahnev & Denison, 2018). For example, we show how different errors in processing the decision context (e.g., undervaluing the high reward stimulus) can be converted into a direct prediction about the amount of bias. Additionally, we have included a few secondary measures here as examples, but this is by no means an exhaustive list of what is possible to pair with the SDT framework. What we propose here are tasks optimised for SDT that could increase our understanding of affective bias identified by previous studies not optimised for this approach (e.g., Aylward, Hales, et al., 2019).

An advantage of being more precise about the sources of bias is that different interventions may be better suited to targeting different sources. This is known as ‘equifinality’, where multiple mechanisms lead to the same endpoint. Specifically, in this case, it may be that all the mechanisms we highlight are important in driving affective bias, but vary from individual to individual. At the same time, different treatments may be better suited to targeting different mechanisms. Antidepressant medication, for example, may target learning mechanisms, whereas psychological therapy targets prior beliefs. Assaying current interventions against the tests we propose here may therefore eventually enable us to match individuals to treatments that will work for them.

As with all methods though, the approach we have outlined does have some limitations. One potential limitation is that while individuals with mood and anxiety disorders demonstrate broad alterations in cognitive performance (Rock et al., 2014), many of the clearest alterations are in executive function and aspects of cognition that are potentially ‘upstream’ of the perceptual processes that SDT is perhaps best suited to model. Moreover, we have restricted our description of the choice context to priors, payoffs, and performance, because these are well integrated with the SDT framework. It is likely other factors that influence the choice context are unable to be captured by SDT (e.g., emotions, social factors, framing). Nevertheless, being precise about potential sources of bias that could emerge from a SDT perspective, and developing tests of these different biases, will ultimately enable us to rule them out before moving on to more complex potential sources of bias. Relatedly, a general concern with laboratory experiments is their ability to generalise to real-world scenarios, yet at the same time be suitable for translational research (Aylward, Hales, et al., 2019; Der-Avakian et al., 2013). This is even more important when applying research in the context of mental health. While the standard signal-detection task might not capture the complexity of our everyday decisions, there is increasingly greater focus in basic science on extending these tasks to more complex, dynamic variants (e.g., Norton et al., 2019) that might help increase the applicability of these tasks.

A third limitation is that for many of the proposed experiments, we are relying on subjective self-reports as secondary measures. While some of these are proving to reveal interesting relationships with mental health (e.g., confidence as shown by Rouault et al., 2018), others are newer or have been developed for the purposes of this article. Though there is increasingly more interest in developing non-introspective measures of beliefs, such as monitoring hand movements (e.g., Dotan, Meyniel, & Dehaene, 2018; Hudson, Maloney, & Landy, 2007; Trommershäuser, Maloney, & Landy, 2008) or eye movements (e.g., Balsdon, Wyart, & Mamassian, 2020; Lempert, Chen, & Fleming, 2015). If self-report methods do not prove useful, these sorts of indirect methods may be necessary to measure beliefs.

Finally, there are also general response biases that arise for reasons such as the experimenter’s instructions (Morgan, Dillenburger, Raphael, & Solomon, 2012), different amounts of effort required to make each of the responses (Hagura, Haggard, & Diedrichsen, 2017), or the Gaussian-noise assumption of the basic SDT model is incorrect (Maloney & Thomas, 1991). However, as these are likely to be consistent across the clinical and control groups, they are less of a concern.

## Summary

In this paper, we have highlighted the advantage of the SDT framework for quantifying suboptimal decision-making and the various underling mechanisms which may drive affective bias in psychiatric disorders. We have also suggested a series of studies that could tease apart which of these mechanisms lead to affective bias using secondary measures and the predictive power of the SDT framework (Ackermann & Landy, 2015; Rahnev & Denison, 2018). The appeal of studying affective bias with low-level perceptual tasks is that such tasks are particularly amenable for testing both humans and animal models (Choi et al., 2016; Scannell & Bosley, 2016). Being more precise about the causes of affective bias in humans may allow us to more precisely test how representative the animal models are and hence bolster the theoretical underpinnings of drug development pipelines for treatments of anxiety and mood disorders.

## Author Summary

We propose applying the Signal Detection Theory framework to understanding the mechanisms behind affective bias in individuals with mood and anxiety disorders. Based on previous explanations of perceptual bias in healthy human observers, we generate five testable hypotheses about the causes of affective bias in clinical populations.
